# Tumor Cellular and Microenvironmental Cues Controlling Invadopodia Formation

**DOI:** 10.3389/fcell.2020.584181

**Published:** 2020-10-15

**Authors:** Ilenia Masi, Valentina Caprara, Anna Bagnato, Laura Rosanò

**Affiliations:** ^1^Unit of Preclinical Models and New Therapeutic Agents, IRCCS – Regina Elena National Cancer Institute, Rome, Italy; ^2^Institute of Molecular Biology and Pathology, CNR, Rome, Italy

**Keywords:** invadopodia, cytoskeleton, cell invasion, metastasis, extracellular matrix, receptors, tumor microenvironment

## Abstract

During the metastatic progression, invading cells might achieve degradation and subsequent invasion into the extracellular matrix (ECM) and the underlying vasculature using invadopodia, F-actin-based and force-supporting protrusive membrane structures, operating focalized proteolysis. Their formation is a dynamic process requiring the combined and synergistic activity of ECM-modifying proteins with cellular receptors, and the interplay with factors from the tumor microenvironment (TME). Significant advances have been made in understanding how invadopodia are assembled and how they progress in degradative protrusions, as well as their disassembly, and the cooperation between cellular signals and ECM conditions governing invadopodia formation and activity, holding promise to translation into the identification of molecular targets for therapeutic interventions. These findings have revealed the existence of biochemical and mechanical interactions not only between the actin cores of invadopodia and specific intracellular structures, including the cell nucleus, the microtubular network, and vesicular trafficking players, but also with elements of the TME, such as stromal cells, ECM components, mechanical forces, and metabolic conditions. These interactions reflect the complexity and intricate regulation of invadopodia and suggest that many aspects of their formation and function remain to be determined. In this review, we will provide a brief description of invadopodia and tackle the most recent findings on their regulation by cellular signaling as well as by inputs from the TME. The identification and interplay between these inputs will offer a deeper mechanistic understanding of cell invasion during the metastatic process and will help the development of more effective therapeutic strategies.

## Introduction

The metastatic cascade is a multistep process characterized by the ability of tumor cells to cross the anatomical barriers, to invade through the surrounding tissues, to reach blood or lymphatic vessels, and to colonize distant organs (Friedl and Wolf, 2010; Lambert et al., 2016). To complete these challenging events, metastasizing cancer cell might adapt to the ever-changing microenvironmental contexts by undergoing reversible changes, often associated with epithelial-to-mesenchymal transition (EMT) and the reverse mesenchymal-to-epithelial transition (MET) (Nieto et al., 2016). In these trans-differentiation processes, cancer cells undergo to change in cell-cell adhesion and polarity, cytoskeletal remodeling, enhanced migratory and invasive abilities. Strictly related to the cell plasticity, in response to mechanical or chemical cues, invading cells might achieve degradation and invasion into the extracellular matrix (ECM), a non-cellular structure comprising the basement membrane and the interstitial matrix, and the underlying vasculature, using focalized proteolysis. One way of delivering matrix proteases and degrading ECM is through invadopodia, F-actin-based protrusive structures coupling adhesive, degradative, and contractile machinery, whose activity represents a key step during cancer invasion (Eddy et al., 2017). The membranes associated with invadopodia contain unique lipid and protein components distinct from the surrounding plasma membrane, while a finely regulated vesicle trafficking to and from the plasma membrane facilitates the invadopodium assembly and function (Hastie and Sherwood, 2016). Crucial regulators of invadopodia include growth factors, and ECM molecular composition, density, organization, and stiffness (Friedl and Wolf, 2010), as well as hypoxia and pH, thus representing structures that may adapt and even interchange in response to the tumor microenvironment (TME) (McNiven, 2013; Di Martino et al., 2016). Recently, significant advances have been made in the understanding invadopodia life cycle. As resulting by genomic and proteomic analysis, as well as by *in vivo* genetic screens, many genes are associated with invadopodia formation, function, and breakdown, including actin regulators, integrins, as well as genes involved in glycolysis, metabolism, protein degradation, chaperone activity, and protein synthesis, although how these players are integrated with different cellular context is still unknown (Attanasio et al., 2011; Hoshino et al., 2013; Lohmer et al., 2016). In this review, we will focus on the regulation of invadopodia by members of signaling pathways as well as by inputs from TME and how their interplay determines a fine regulation of invadopodia.

## General Features of Invadopodia

Cell motility is a tightly coordinated multistep process used by different cells to reach the different sites of action, essential in physiological processes, such as embryonic morphogenesis, immune surveillance, tissue repair, but guiding cancer invasion and metastasis when aberrantly regulated (Condeelis and Segall, 2003). This implies the formation of extensions of cell membrane such as filopodia, lamellipodia, podosomes/invadopodia, based on their morphological, structural, and functional characteristics (Ridley, 2011). Each of these structures uniquely contributes to migration depending on the specific cellular and tissues context and the actin dynamics are the result of a concerted regulation of parameters governing the assembly, stability, and organization of actin filaments by a specific set of proteins. In brief, lamellipodia are flat cellular protrusions located at the leading edge of the migrating cells, where actin is organized in an orthogonal array of branched filaments, maintained in fast treadmilling by a set of regulatory proteins (cofilin, capping proteins, profilin), determining the protrusive forces pulling cells through the tissues (Machesky, 2008). Filopodia are thin, finger-like protrusions beyond the leading edge of protruding lamellipodia at of the migrating cells, with the characteristics to explore the cell’s surroundings (Jacquemet et al., 2015). Distinct from filopodia and lamellipodia, matrix-degrading protrusions, called invadopodia in cancer cells and podosomes in normal cells, collectively invadosome, are complex subcellular structures comprised of a dense filamentous (F)-actin core containing actin-regulating proteins, surrounded by proteins involved in regulation, adhesion and scaffolding (Schachtner et al., 2013; Eddy et al., 2017). Unlike podosomes which are very short-lived and not protrusive, invadopodia can last for hours as long protrusive structures, assembled in a highly orchestrated manner and observed as individual dots or linear structures (Artym et al., 2006). These structures are involved in the cell-ECM interactions, but their specific characteristic resides in the proteolytic activity. Each invadopodia (non-degradative precursor) is initially composed of an F-actin core enriched in actin-regulating proteins, including cortactin, cofilin, neural Wiskott–Aldrich syndrome protein (N-WASP) and Arp2/3, then surrounded by a ring of actin regulatory and adhesive molecules, such as integrins, talin, vinculin, and paxillin. The recruitment of the adaptor protein tyrosine kinase substrate with five SH3 domains (TKS5) anchors the precursor to the membrane phosphoinositide PI(3,4)P2 via its PX domain (Linder et al., 2011; Beaty and Condeelis, 2014; Eddy et al., 2017). The invadopodia elongation and stabilization, driven by actin polymerization and facilitated by the recruitment of microtubules and intermediate filaments, start the maturation stage, which is completed with the activation of the secretory machinery and recruitment of proteases degrading the ECM (Artym et al., 2006; Oser and Condeelis, 2009; Schoumacher et al., 2010; Linder et al., 2011; Sharma et al., 2013). The main proteases at invadopodia include metalloproteinases (MMPs), both secreted and membrane-tethered, such as MMP-2, MMP-9, and MT1-MMP, the ADAM family members, membrane-bound serine protease and the urokinase plasminogen activator receptor (uPAR) (Mrkonjic et al., 2017). Among these, MT1-MMP is strongly enriched at invadopodia and represents a central player of invadopodia-mediated ECM degradation (Castro-Castro et al., 2016). Although inputs derived from cancer cells or TME trigger the activation of specific signaling pathways controlling invadopodia formation, common pathways are recognized as regulators of actin dynamics at invadopodia, especially the cofilin pathway and the integrated activity of Rho family GTPases.

Cofilin localizes at invadopodia and it is involved in their formation and stability as well as their maturation. Cofilin acts by depolymerizing actin filaments to supply a pool of actin monomers or by severing actin filaments to create free barbed ends, both necessary for actin polymerization (Yamaguchi et al., 2005; Oser et al., 2009). The primary on/off regulation proceeds by blocking its activity through binding to either PI (4,5)P2 or cortactin, or by blocking cofilin’s ability to bind to actin via serine phosphorylation. Specific kinases involved in phosphorylation/inactivation of cofilin include the Lim and the Tes family kinases, while phosphatases dephosphorylating and activating cofilin include slingshot, chronophin as well as PP1, PP2A, and PP2B (Oser et al., 2009). The activity of cofilin in invadopodia might be also regulated by cortactin, a multi-domain scaffolding protein activating the Arp2/3 complex, and binding actin filaments to stabilize them (Schnoor et al., 2018). In resting conditions, cortactin binds cofilin and inhibits its severing activity, while tyrosine phosphorylation of cortactin decreases cortactin/cofilin interactions favoring actin polymerization. Therefore, since common kinases known to phosphorylate cortactin include downstream effectors for several cell receptors, such as Src, Fer, Arg, and Abl, it is reasonable to speculate that cofilin signaling represents a converging point for invadopodia regulation by multiple mechanisms and factors (Oser et al., 2009).

The activity of Rho family GTPase is recognized as a convergent and common pathway from different inputs and an essential regulator of actin dynamics at invadopodia in each specific step. While Cdc42 alone or in cooperation with Rac1 participates only during the precursor assembly (Nakahara et al., 2003; Yamaguchi et al., 2005; Ayala et al., 2009; Di Martino et al., 2014; Lin et al., 2014; Kedziora et al., 2016; Ren et al., 2018), RhoA alone or in cooperation with Cdc42 is specifically involved in invadopodia maturation (Sakurai-Yageta et al., 2008; Hoshino et al., 2009; Daubon et al., 2012; Hwang et al., 2016; Yan et al., 2018). More recently, RhoC is emerging as a crucial coordinator of cofilin-dependent actin polymerization to the core of invadopodia, associated with focused ECM degradation and cell invasion (Bravo-Cordero et al., 2011; Pignatelli et al., 2012; Semprucci et al., 2016; Di Modugno et al., 2018). Regarding Rac1 and the related RhoG GTPase, the effects seem to be cell- and context-dependent, since their activity is required for invadopodia formation in some cancer cell lines, while they regulate invadopodia disassembly in some others (Harper et al., 2010; Nascimento et al., 2011; Kwiatkowska et al., 2012; Pignatelli et al., 2012; Lin et al., 2014; Moshfegh et al., 2015; Goicoechea et al., 2017). Similarly, Rac3 activity, which integrates adhesion signaling and ECM degradation, is confined to a ring around actively degrading invadopodia for the surface presentation of MT1-MMP (Donnelly et al., 2017; Rosenberg et al., 2017). Of course, the tight spatiotemporal pattern of Rho GTPase activation at invadopodia requires an interplay between guanine exchange factors (GEFs), leading to the activation of GTPases by stabilizing the GTP-bound form, and GTPase activating proteins (GAPs), which inhibit them. In this context, many GEFs are considered as regulators of invadopodia, like Vav1, βPIX, Trio, DOCK1, ARHGEF26, PDZ-RhoGEF, p190GEF, as well as GAPs, such as RacGAP1, p190GAP, although the interplay between inputs and Rho GTPase activity in a specific cellular context remains to be fully characterized (Lawson and Ridley, 2018).

New features are now emerging, as the importance of cell cycle status and cell cycle regulators in determining invadopodia (Bayarmagnai et al., 2019). Specifically, it has been shown that invadopodia function is enhanced in the G1 phase of the cell cycle *in vitro* and *in vivo* when the expression of invadopodia markers is elevated, and cells are more prone to degrade ECM. Of note, the cell cycle regulator p27kip1 localizes to the sites of invadopodia assembly, determining faster turnover and increased ECM degradation (Bayarmagnai et al., 2019). From a translational point of view, these findings suggest the importance to consider that the use of anti-proliferative drugs arresting cancer cells in G1 might result in higher invasion and metastasis by supporting invadopodia activity (Bayarmagnai et al., 2019).

## Cellular Signals Affecting Invadopodia Formation and Function

### Receptor Signaling Pathways

**FIGURE 1:**
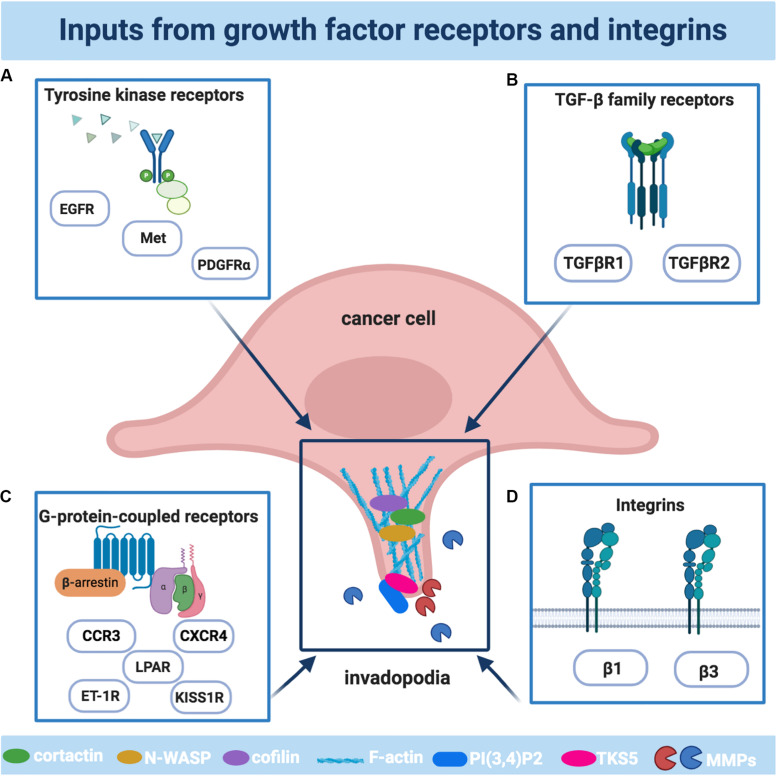
The main receptors involved in the formation and activities of invadopodia. Schematic illustration of the main cellular receptors driving invadopodia in cancer cells: **(A)** The family of tyrosine kinase receptors includes epidermal growth factor receptor (EGFR), platelet-derived growth factor receptor α (PDGFRα), Tyrosine-Protein Kinase Met (Met); **(B)** The family of G-protein-coupled receptors (GPCR) includes lysophosphatidic acid receptors (LPAR), endothelin-1 receptors (ET-1R), kisspeptin receptors (KISS1R), C-X-C Motif Chemokine Receptor 4 (CXCR4), C-C Motif Chemokine Receptor 3 (CCR3); **(C)** The transforming growth factor β (TGFβ) receptors family includes TGFβR1 and TGFβR2; **(D)** The family of integrins includes the β1 and β3 subunits. The plot was created using BioRender (app.biorender.com).

The activation of receptors by their ligands represents the means of communication between tumor and stroma, used by tumor cells to operate invadopodia-dependent cell invasion. Their overexpression or overactivation can result in a state of continual signaling, through an autocrine or paracrine way, triggering the activation of intracellular signal cascades converging on the common invadopodia-related pathways, including Src, phosphoinositide 3-kinase (PI3K), and Rho family GTPases. Therefore, the ability of tumor cells to sense and migrate in response to receptor signals is required to restrict actin remodeling events and to coordinate in time and space cytoskeletal signaling proteins, eliciting invasive protrusions. Here we describe the main receptor families involved in invadopodia (Figure 1).

#### Tyrosine Kinase Receptors

Members of the large family of receptors with tyrosine kinase activity (RTK) are key regulators of cancer cell growth, proliferation, and survival, as well as invasion and metastasis (Du and Lovly, 2018). There is a large body of works indicating that the activation of some RTKs operates to trigger signaling events, which can even be integrated with signals from TME, having invadopodia formation as a common endpoint. The epidermal growth factor receptor (EGFR), eliciting a ligand-dependent response in different cancer cell lines, is considered a master regulator of invadopodia. The main pathways guided by EGF are related to enhanced actin polymerization, through N-WASP-Arp2/3/cofilin pathway, during invadopodia assembly (Yamaguchi et al., 2005) or invadopodia maturation, through a mechanism involving Src and cortactin phosphorylation (Kimura et al., 2010; Mader et al., 2011; Makowiecka et al., 2016). EGFR signaling in invadopodia might be also sustained by a high level of heparin-binding (HB)-EGF, which is synthesized as pro-HB-EGF and subsequently cleaved by ADAM-12 to release a soluble form binding to EGFR, providing an advantage for cancer cells to intravasate, invade and metastasize (Zhou et al., 2014). A further amplification mechanism is provided by the localization of proteases operating the ectodomain shedding of EGFR ligands, increasing their availability, thus suggesting that invadopodia-related proteases may provide spatiotemporal control of growth factors promoting the invasive and metastatic potential of cancer cells, in addition to their role of proteolyzing ECM (Albrechtsen et al., 2011). EGFR signaling might promote invadopodia also via crosstalk with other cell surface receptors, as CD44 or CD147, or kisspeptin receptor (KISS1R), generating a pool of invadopodia-forming signals (Bourguignon et al., 1998; Grass et al., 2013; Goertzen et al., 2016).

Platelet-derived growth factor receptor α (PDGFα) is an important effector of invadopodia in aggressive human breast tumors (Govaere et al., 2017), and represents a central mediator in response to EMT-inducing signals, such as related transcription factors (Ekpe-Adewuyi et al., 2016). In particular, the transcriptional induction of PDGFRα and downstream activation of Src driven by the EMT-related transcription factor Twist1, is essential for invadopodia formation and matrix degradation, as well as *in vivo* metastasis, indicating that the reactivation of developmental machinery, such as the EMT, in tumor metastasis might act in cooperation via invadopodia (Eckert et al., 2011). In pancreatic cancer, β-catenin activation, coupled with K-ras mutation and p53 loss, activates an autocrine PDGF signaling with highly invasive properties of tumor cells, capable to form invadopodia and to digest ECM (Kuo et al., 2019). Finally, signals from an activated tyrosine kinase receptor Met might also increase invadopodia biogenesis in basal-like breast and gastric carcinoma cells, dependent on tyrosine phosphorylation of cortactin (Rajadurai et al., 2012).

#### G-Protein-Coupled Receptors

G protein-coupled receptors (GPCRs) are heptahelical membrane proteins, which classically transmit the signal via the activation of heterotrimeric G proteins (Gurevich and Gurevich, 2019). They are activated by small peptides, hormones, and chemokines, triggering their specific downstream signaling events regulating cell shape changes, altered cell adhesion, actin remodeling, and driving cell migration (Cotton and Claing, 2009). Agonist-activated GPCRs act as GEFs for heterotrimeric G proteins, facilitating the release of GDP bound to the α-subunit of inactive heterotrimer, which subsequently binds GTP. Then Gα subunit dissociates from the GPCR and Gβγ dimer, and both GTP-liganded α-subunit and released Gβγ activate or inhibit various signaling pathways (Peterson and Luttrell, 2017). The signaling of most GPCRs via G proteins is terminated by the phosphorylation of active receptor by specific GPCR kinases (GRKs) and subsequent binding of β-arrestin (β-arr) proteins, β-arr1 and -2, that selectively recognize active phosphorylated receptors. The β-arr/GPCR complex acts as a scaffold facilitating different branches of signaling, and several findings have identified β-arrs as critical regulators of cytoskeleton remodeling and cell motility (DeFea, 2013).

In the GPCR family, the lysophosphatidic acid (LPA) receptor has been demonstrated to operate invadopodia formation. For instance, the secretion of Autotaxin, a major enzyme involved in the production of LPA, drives the activation of LPA4 receptor and subsequent activation Rap1/Rac1 signaling, acting as a strong inducer of invadopodia, and correlating with the ability of fibrosarcoma cells to invade and metastasize (Harper et al., 2010). In ovarian cancer cells, LPA signaling causes the translocation of Gαi2 into the invadopodia, forming a large molecular complex with β-pix and Src, regulating their formation (Ward et al., 2014). A similar mechanism has been demonstrated in melanoma cells through balancing Cdc42/RhoA activity (Kedziora et al., 2016). According to the idea that the same receptor might activate different signaling pathways in different tumor histotypes, in prostate cancer cells, LPA increases functional invadopodia formation through RhoA and NF-κB, controlling osteolytic metastases (Hwang et al., 2016).

In the same family, acting on the endothelin type A (ET_A_) or type B (ET_B_) receptor, the small peptide endothelin-1 (ET-1), recognized as a critical regulator of different human cancers (Rosanò et al., 2013), provides a signal input for shaping invasive protrusions with efficient matrix degradation during cancer invasion and metastases. The first evidence of the role of ET-1 in invadopodia formation has been highlighted in human melanoma cells, where ET-1 through Gi activates Cdc42 GTPase while decreasing RhoA (Kedziora et al., 2016). In ovarian cancer, the formation of invadopodia and their proteolytic activity is achieved by a combination of players and signaling molecules that make part of integrated molecular complexes coordinated by the β-arr1 (Rosanò and Bagnato, 2016, 2019). Upon ET-1/ET_A_R stimulus, β-arr1 interactions determine the convergence and activation/inhibition of specific signals for invadopodia, functioning as a dynamic molecular scaffold with the ability to interact with an ever-expanding list of non-GPCR protein partners. ET-1-driven spatial and temporal coordination of actin polymerization at invadopodia implies the coordination of the Rho GTPase and their regulators (Rosanò and Bagnato, 2019). RhoC is the main Rho GTPase regulated by β-arr1 through the interaction with PDZ-RhoGEF (Semprucci et al., 2016). At the same time, in the ET-1-dependent manner, β-arr1 links the integrin-related protein IQ-domain GTPase-activating protein 1 (IQGAP1) and RacGAP1, assembling them into a functional unit to promote Rac1 inhibition and concomitant RhoC activation (Chellini et al., 2019). The activity and the spatial distribution of RhoC represent a critical route by which tumor cells control the recruitments of cortactin, TKS5, and matrix proteases in the formation of invadopodia precursors, and the spatial restriction of cofilin activity, starting the maturation process. Although previous findings showed the involvement of β-arr1 in cofilin regulation (DeFea, 2013), new data shed light into the ability of β-arr1 in participating to dynamically define the amount and distribution of actin barbed ends to regulate invasive protrusion, by confining cofilin activity within the core of invadopodia. Moreover, β-arr1-associated molecular complexes in invadopodia involve the presence of hMENA and the invasive isoform hMENAΔv6, members of the ENA/VASP family, known to regulate the actin-based motility of various cell types (Di Modugno et al., 2012, 2018). The formation of a signaling platform driven by ET-1, containing β-arr1/hMENA/hMENAΔv6/PDZ-RhoGEF and converging on the RhoC pathway, supports pericellular matrix degradation and confers also a fitness advantage to tumor cells to breach the endothelial barrier and engage them in the transendothelial migration process (Di Modugno et al., 2018). Therefore, ET_A_R/β-arr1 core is necessary to assemble elements for invadopodia formation, as cortactin and TSK5, as well as regulating invadopodia maturation, disclosing so far unexpected involvement of β-arr1 capable of promoting actin assembly to form invadopodia, and regulating the release of specific proteinases at invadopodia, hence enabling ovarian cancer cells to invade and metastasize (Rosanò and Bagnato, 2019).

In breast cancer, the activation of the GPCR KISS1R stimulates invadopodia formation via β-arr2 and ERK1/2-dependent mechanisms, involving the crosstalk with EGFR (Goertzen et al., 2016).

Chemokine receptors, belonging to the GPCR family, are considered key factors promoting the invasive program of metastatic cancer, and many findings highlighted their role in invadopodia (Balkwill, 2003). In breast cancer cells, the chemokine receptor CXCR4 and its ligand SDF1α activate Abl kinase, regulating MT1-MMP trafficking and recruitment to invadopodia and matrix degradation activity (Smith-Pearson et al., 2010). In glioma cells, the autocrine CXCL12/CXCR4 signaling axis regulates invadopodia formation, ECM degradation, and cell invasion, by inducing cortactin tyrosine phosphorylation through a mechanism involving Arg (Chen et al., 2020). In lung cancer, CCL7 through its receptor CCR3 regulates MMP-9 transport to the invadopodia, thereby promoting the ability of invadopodia to degrade collagen and invade ECM, favoring metastases, through RhoA activation (Qi et al., 2020).

#### TGF-β Family Receptors

TGF-β family members bind and signal through paired transmembrane protein kinases, type I and type II receptors, and different ectodomain combinations enable selective or specific binding of TGF-β family ligands and ligand-induced activation of signaling (David and Massagué, 2018). TGF-β, the prototype ligand for this receptor family, has been shown to promote ECM degradation and invasion through the formation of invadopodia (Mandal et al., 2008; Makowiecka et al., 2016). During EMT, the interaction with components of focal adhesions, such as Hic-5, plays a bifunctional role in coordinating FAK-Src activity and downstream both Rac1- and RhoC-ROCK-dependent matrix degradation after TGF-β stimulation (Pignatelli et al., 2012). Similarly, TGF-β might control EMT and invadopodia via Transgelin, an actin-binding protein that affects the dynamics of the actin cytoskeleton, further confirming the association between EMT and invadopodia during tumor spreading (Chen Z. et al., 2019). In this context, cells exposed to TGF-β accelerate the activation of signal pathways enhancing β3 integrin expression (Peláez et al., 2017). In breast cancer, TGF-β-driven invadopodia requires the involvement of lipoma-preferred partner (LPP) as well as SHC adaptor protein (SHCA) for an efficient metastatic process (Ngan et al., 2017; Kiepas et al., 2020).

#### Integrins

The integrins, among the transmembrane receptors connecting cell cytoskeleton to ECM components, interact via their extracellular domains to specific sequence motifs present in proteins such as fibronectin, collagen and other ECM proteins, and are connected to the actin cytoskeleton via their cytoplasmic tails, and by a complex of multi-protein complex integrin adhesome, comprising adaptor, scaffold, and signaling proteins (Cooper and Giancotti, 2019). Although for many times the role of integrins in invadopodia was a bit controversial, recently it has been demonstrated that the gradual ECM breakdown and release of ligands for integrin receptors might increase cancer cell invasion and intravasation via invadopodia formation. Moreover, specific integrin subunits are recruited to invadopodia after the initial stage to form an adhesion ring (Peláez et al., 2019). This adhesive domain regulates invadopodia formation by activation of specific Rho GTPase family members and various kinases, together with the recruitment of adhesion molecules, in a tissue-specific way. Besides their role in the adhesive domain, integrins exert a necessary function in the invadopodia maturation, generating also the forces required for ECM degradation, through interaction with talin2 (Qi et al., 2016). The best-characterized integrin subunit in invadopodia is β1, transducing signaling from fibronectin, laminin or collagen, at different stages of invadopodia formation, cooperating also with other transmembrane receptors, like CD44 or EGFR (Nakahara et al., 1996, 1998; Hauck et al., 2002; Nascimento et al., 2011; Williams and Coppolino, 2014; Siqueira et al., 2016). The activity of β1 integrin includes regulation of tyrosine phosphorylation of cortactin, through a direct interaction with Arg (Beaty et al., 2013), or with Mena (Oser et al., 2010; Mader et al., 2011; Gupton et al., 2012; Beaty et al., 2013). β1 integrin is also important in the recruitment of talin and moesin, as well as ezrin, to the invadopodia core and the attachment of NHE1, a sodium-hydrogen antiporter controlling the lipid raft signalosome driving invadopodia (Antelmi et al., 2013). One of the most important effects of β1 integrin is to promote focalized recruitment of MT1-MMP, modulating the vesicle traffic and release at invadopodia, as well as the MT1-MMP internalization (Grafinger et al., 2020). Together with β1 integrin, the focal adhesion protein integrin-linked kinase (ILK) regulates invadopodia activity by recruiting the scaffold protein IQGAP and by inducing MT1-MMP activity and matrix degradation (Branch et al., 2012; Chellini et al., 2019). Using an interactive analysis based on time-lapse microscopy and mathematical modeling, it has been demonstrated that cancer cells oscillate between invadopodia/degradation and cell migration phenotypes (Pourfarhangi et al., 2018). Interestingly, invadopodia state can be removed by partial β1-integrin inhibition or enhanced cross-linked, suggesting a therapeutic window in which it might be possible of targeting invadopodia via ECM-modulation treatments (Pourfarhangi et al., 2018). The role of β3 integrin in invadopodia formation is controversial. Although some authors have proposed that this integrin is not implicated in invadopodia development, β3 overexpression correlates with invadopodia formation and matrix degradation in renal carcinoma, sarcoma, breast and lung cancer cells, and glioblastoma cells (Knowles et al., 2013). Other integrin subunits involved in invadopodia formation and functions include β 5-, α 5-, α3- and αV-integrins (Yan et al., 2018).

Understanding the molecular mechanisms driven by cellular signals adds new insight into the multifaceted role of these receptors in the process of metastasis in general, and invadopodia in particular, and might indicate new strategies for more selective targeting of these receptors, therefore potentially providing a therapeutic approach for preventing metastatic dissemination.

### Signals From the Tumor Microenvironment

**FIGURE 2:**
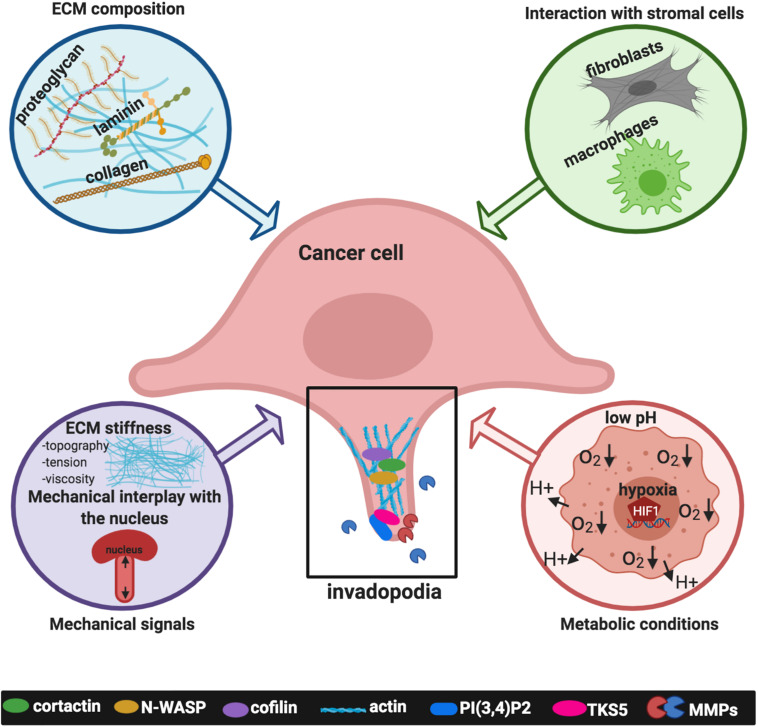
Signals deriving from the tumor microenvironment (TME) affecting invadopodia formation and activity. Inputs derived from the TME include the extracellular matrix (ECM) composition, such as collagens, laminin, fibronectin; the interaction with stromal cells, specifically activated fibroblasts, and macrophages; mechanical signals such as matrix stiffness, topography, tension, viscosity and the mechanical interplay with the nucleus; metabolic conditions typical of cancer cells, such as extracellular acidosis and intracellular low tensions oxygen (hypoxia). The plot was created using BioRender (app.biorender.com).

Over the past few decades, different omics approaches strongly defined that tumor progression and dissemination critically depend on a permissive TME, composed of non-cancerous cells, including fibroblasts, immune and inflammatory cells, and cells forming the tumor vasculature, as well as acellular components surrounding and interacting with tumor cells (Quail and Joyce, 2013). Cancer cells reside in a harsh TME together with different stromal cell types and the communication between tumor cells and heterogeneous stromal components contributes to tumor progression while affecting therapeutic responses. The tumor ECM is different from normal tissue, in tumors of different stages, in primary tumors from the secondary tumors, and is characterized by other peculiar parameters, such as low oxygenation levels and pH. All these characteristics participate to orchestrate cancer cell processes during tumor progression linked also to cell invasion. Alterations of the ECM biochemical or mechanical properties, such as composition, geometry, alignment, and stiffness, as well as the porosity, determine the rates and routes of metastatic dissemination (Yuzhalin et al., 2018). In this context, emerging findings indicate that cancer-cell-derived matrisome proteins can upregulate invadopodia, hence promoting metastasis, and suggest that identifying ECM regulators of dynamic matrisome licensing cancer progression and metastasis, can make them potential targets for cancer therapy (Tian et al., 2020). In this scenario, here we summarize relevant inputs derived from the TME regulating invadopodia (Figure 2).

#### ECM Composition

The ECM components include several proteins, such as collagens, laminins, fibronectin, or heparin sulfate proteoglycans, and many others. ECM proteins are produced by both stromal and tumor cells; however, cancer-associated fibroblasts (CAFs) are the main source for synthesis, secretion, and assembly of the ECM components, and hence critically involved in the modification of the ECM composition and organization. Besides their architectural role in providing an anchorage and support to the surrounding cells, ECM proteins transmit signaling which is interpreted and transduced by specific cell receptors (Nazemi and Rainero, 2020). Although each component of ECM plays a specific role in cancer progression, the role of collagen stands out, influencing invasive behavior through integrins, TRKs, discoidin domain receptors (DDRs), and other signaling pathways, a phenomenon which can be amplified in hypoxic conditions. Also, the interaction of collagen with other ECM molecules, such as fibronectin, laminin, and MMPs, influences cancer cell activity (Baghban et al., 2020). Collagen I, collagen IV α1 and collagen XIII α1 can induce linear invadopodia, both dependently or independently of integrins, but in some cases involving DDR1 (Juin et al., 2012, 2014; Miyake et al., 2017; Yan et al., 2018). In the family of laminins, it has been reported that AG73 and C16 laminin-111-derived peptides induce invadopodia formation in adenoid cystic carcinoma, fibrosarcoma, and bladder carcinoma cells (Nascimento et al., 2011; Siqueira et al., 2016).

#### Mechanical Signals

Besides its biochemical composition, biophysical characteristics of the ECM, including topography, stiffness/rigidity, molecular density, and tension, are strongly subject to remodeling under the influence of tumor stroma and cancer cells. Cancer cells adapt to mechanical alterations of the local stroma by transducing them into biochemical signaling events that guide and reinforce the invasion of cancer cells (Menon and Beningo, 2011; Mierke, 2019). Hence, more active invadopodia were formed upon mechanical stimulation (Alexander et al., 2008; Albiges-Rizo et al., 2009; Gasparski et al., 2017). The best-characterized mechanical cue in invadopodia-related function is linked to the compliance of the ECM, as enhanced formation and activity of invadopodia has been observed on stiff ECM compared to soft ECM (Alexander et al., 2008; Parekh et al., 2011; Aung et al., 2014; Berger et al., 2020). Depending on the means of stimulation, the same cell can organize its actin cytoskeleton into classical dot-like or linear invadopodia. Independently of growth factor stimulation, the dense network of fibrillar collagen, extensive deposition of fibrillar collagens in the tumor ECM as observed in advanced stages of cancers, is a crucial inducer of invadopodia in both tumor cells and stromal fibroblasts, which proteolytically degrade and remodel the surrounding ECM, governed by a complex integrin signaling network (Artym et al., 2015). However, some cancer cells preferentially form linear actin structures on fibrillar collagen I, characterized by the appearance of individual dots, without adhesion ring proteins (Juin et al., 2012, 2014; Schachtner et al., 2013; Di Martino et al., 2015). Other studies implicate tugging forces on the ECM fibers as a specific mechanical signal for the maturation of invadopodia and the increase in their length (Gasparski et al., 2017). Of note, while it has been long appreciated that tumor tissues are stiffer than more normal tissues, recently it has been highlighted that the tumor tissues are characterized by elevated viscosity and that cancer cells might generate forces to migrate through these confined matrices (Wisdom et al., 2018). In this context, invadopodia can generate protrusive, degradative, and contractile forces to initiate the matrix opening and physically expand the pores, where the length of the channel dictates the speed and distance of cell migration, followed by a protease-independent invasion through confining plastic matrices (Wisdom et al., 2018). These findings strongly support the idea that invadopodia are utilized in both protease-dependent and protease-independent migration, as two extremes of the mesenchymal migration, and that the mechanical plasticity of cancer cells linked to invadopodia permits that invasive cells can bypass the physical constraints (Wisdom et al., 2018). Recently, a new paradigm has been uncovered, where MT1-MMP acts both as an initiator and executor player of invadopodia and cell invasion in a type I collagen-rich ECM. Indeed, MT1-MMP might direct invadopodia assembly, favoring TKS5 recruitment and formation of mature invadopodia, while MT1-MMP proteolytic activity contributes to invadopodia expansion and collagen remodeling, by promoting matrix pore enlargement to facilitate tumor-cell invasion (Ferrari et al., 2019). In addition to ECM properties, the compliance of cells acts as a determinant of cell plasticity and as an inducer of invadopodia. Indeed, while cell cytoplasm is readily deformable, the nucleus is stiffer than the cytoplasm and this determines a nuclear rigidity and barrier deformability, dependent on lamin A, thus representing a factor limiting the invasion. Invadopodia are preferentially formed under the nucleus and their connection with the nucleus could have a role in the transmitting forces required for invadopodia to protrude through an ECM (Revach et al., 2015). Dissecting the mechanobiology connected to invadopodia, it has been proven an important mechanism that overcomes the limitation of cancer cell migration in constricting pores operated by nuclear stiffness. This mechanism depends on a specific linkage of the nucleus to the microtubule-centrosome network generating of forwarding nucleus pulling force, required for MT1-MMP endosome positioning and targeted delivery of MT1-MMP to invadopodia, leading to enlarged matrix pores and permitting migration, and avoiding nuclear deformations, loss of nuclear envelop integrity and DNA damage (Infante et al., 2018). Moreover, findings measuring the pushing forces generated by the invadopodia through the ECM provide a new perspective in which mature degradative invadopodia exert to ECM mechanical higher forces than non-degrading ones, thus enhancing cancer invasion (Dalaka et al., 2020).

These data suggest that invadopodia are plastic structures, highly adaptable to the matrix microenvironment and acting as matrix mechanosensory, thus reflecting the ability of the cells to exploit and invade different types of tissues and matrices and that mechanical stimulation may accelerate the rate of the maturation process enhancing cell invasion.

#### Interactions Between Cancer and Stromal Cells

In the tumor microenvironment, the reciprocal physical interaction between cancer and surrounding stromal cells represents a factor promoting invadopodia. CAFs play an important role in tumorigenesis, and their interaction with tumor cells occurs at several interfaces, including the production of ECM proteins, the release of nutrients, and cytokines that facilitate the metastatic progression. The complex and mutualistic interactions between tumor cells and neighboring fibroblasts are critically involved in matrix-degrading proteases secretion and ECM remodeling. Fibroblasts itself can degrade matrix independent of invadopodia, supporting invasion indirectly through mechanical regulation, or serving as “leader” cells (Brentnall et al., 2012; Goicoechea et al., 2014). In the cross-talk between tumor cells and fibroblasts, pancreatic cancer cells can induce, in a paracrine way, the expression of the cytoskeletal-related protein paladin and the conversion of fibroblasts into CAFs. In this process, the activation of Cdc42 and the formation of invadopodia generate migratory tracks through the ECM facilitating cells invasion (Goicoechea et al., 2014). In the same tumor type, the interaction of pancreatic cancer cells with pancreatic stellate cells, a major component of the dense stroma characterizing this tumor, results in their differentiation in CAFs, ECM remodeling, and the secretion of cytokines, hence accelerating invadopodia development and cell invasion (Hwang et al., 2019). Similarly, in Extramammary Paget’s disease (EMPD), a major life-threatening skin cancer, TGF-β produced by cancer cells upregulates podoplanin expression in peritumoral basal keratinocytes, mimicking the invasive front of squamous cell carcinoma and supporting tumor cell invasion via invadopodia (Cho et al., 2017).

Metastasis-associated macrophages (TAMs) are other key elements of the TME that significantly affect cancer cell motility and metastatic behavior. Throughout the metastatic cascade, a subset of TAMs accumulates within metastatic sites and the interaction with cancer cells allows them to invade, intravasate into the blood vessels and extravasate into secondary sites, by producing factors fueling cancer invasion and metastasis (Hanahan and Coussens, 2012). Several studies characterizing the mechanisms of tumor cell-macrophage interactions in cancer cell motility showed that the crosstalk between tumor cells and macrophages promotes invadopodia (Yamaguchi et al., 2006; Pignatelli et al., 2014, 2016; Roh-Johnson et al., 2014). In particular, the physical heterotypic contact between macrophages and breast cancer cells activates the RhoA pathway, resulting in increased invadopodium formation in tumor cells at blood vessels. Moreover, the direct contact between macrophages and breast cancer cells promotes MenaINV expression, causing sensitization of tumor cells to growth factor signals (Eddy et al., 2017) and tumor cell intravasation across an endothelial barrier (Pignatelli et al., 2016). A positive feedback paracrine loop between macrophages and cancer cells has been reported in head and neck squamous cell carcinoma. Indeed, cancer cells educate monocytes into M2-like macrophages by releasing C-C motif chemokine ligand 2 which in turn secrete EGF, hence increasing cancer cell motility by mean of invadopodia formation, facilitating tumor local invasion and distant metastasis (Gao et al., 2016).

These findings underscore the importance of fully understanding the contributions of the crosstalk between stromal cells and cancer cells in the tumor microenvironment, underlying the molecular and physical mechanisms regulating matrix remodeling, and invadopodia.

#### Metabolic Conditions

Cancer and stromal cells in TME are immersed in the metabolic conditions characterized by acidosis and low-tension oxygen, known as hypoxia, both considered central issues in tumor metastasis since in these conditions cancer cells have a higher tendency to metastasize (LaGory and Giaccia, 2016). Indeed, in most solid tumors, the rapid tumor growth can outpace their available blood supply with the occurrence of hypoxia. In response to hypoxia, a change in the gene expression pattern of cancer cells is produced, and hypoxia-inducible factors (HIFs) are the major transcriptional regulators in response to hypoxia, which consists of an oxygen-regulated HIF-α subunit (HIF-1α or HIF-2α) dimerizing with HIF-1β, involved in the transcription of genes strongly correlated with tumor growth, angiogenesis, and metastasis. Several findings demonstrated that under hypoxia pressure, cancer cells develop invasive capacities through invadopodia formation and activity (Arsenault et al., 2013; Harper et al., 2018; Yan et al., 2020). For instance, both HIF-1α and HIF-2α can regulate the expression of growth factors and receptors promoting invadopodia, as shown for PDGFRα, directly or dependent by Twist (Eckert et al., 2011; Hanna et al., 2013). Notch is another effector of hypoxia-dependent invadopodia formation in different cancer cells. Indeed, under hypoxia, Notch upregulates the activity of ADAM12 and downstream the shedding of HB-EGF, thus amplifying EGFR-dependent signaling (Díaz et al., 2013). An additional mechanism by which hypoxia might regulate invadopodia is linked to the enhanced activity HDAC6, a member of the histone deacetylase family. HDAC6 might regulate the acetylated level of tubulin and cortactin (Rey et al., 2011). Moreover, hypoxia might enhance HDAC6 tubulin deacetylase activity by upregulating EGFR (Arsenault et al., 2013). A direct link between hypoxia and LPA signaling for invadopodia formation and metastasis has been established. Under hypoxic conditions, LPA1 establishes Src-mediated crosstalk with EGFR, increasing the ability of cells to produce invadopodia (Harper et al., 2018). Moreover, hypoxia-dependent invadopodia regulation is related also to the ability to upregulate molecules involved in cytoskeleton remodeling, as observed for HIF-1α-dependent transcription of β-PIX (Hashim et al., 2013), N-WASP (Salvi and Thanabalu, 2016) or CSRP2, an actin-bundling protein (Hoffmann et al., 2018), or structural components of lipid rafts required for invadopodia formation and protease recruitment, such as caveolin-1 (Wang et al., 2012). Cellular adaptive program triggered by hypoxia via HIF-1α to regulate invadopodia includes also the expression of pH regulators, generating extracellular acidosis and contributing to effective matrix cleavage, through direct or indirect mechanisms (Busco et al., 2010; Lucien et al., 2011; Magalhaes et al., 2011; Brisson et al., 2013). For instance, carbonic anhydrase IX (CAIX), a HIF-1α-induced pH regulating enzyme, might be localized within invadopodia, coordinating activities of both MT1-MMP and actin-regulating proteins, essential for invadopodia elongation, ECM degradation and cell invasion (Swayampakula et al., 2017; Debreova et al., 2019). In addition, the acidification of the extracellular space as well as the increased intracellular pH by NHE1 drives invadopodia by controlling activation of proteases and disrupting the inhibitory interaction between cortactin and cofilin, thus allowing to cofilin-dependent actin polymerization and matrix degradation (Denker et al., 2000; Busco et al., 2010; Magalhaes et al., 2011; Antelmi et al., 2013; Beaty et al., 2014; Greco et al., 2014). These findings strongly indicate the need to dissect more in-depth how hypoxia and the extracellular acidosis act in determining the ability of cancer cells to form invadopodia, cross the ECM and initiate invasion in a cell-autonomous as well as in a non-cell-autonomous manner, further pointing to hypoxia as well as metabolic conditions as targets for therapeutic approaches. In the last years, it has been evidenced that invadopodia-related factors, such as kinase signaling, actin cytoskeleton regulators or proteases, are regulated by calcium (Ca^2+^) signaling, thus representing an interesting actor in invadopodia field and a potential therapeutic target (Iamshanova et al., 2017). Basically, in invadopodia, Ca2 + influx is required for the focal degradation of ECM through the upregulation of proteases, like MMPs and cathepsins (Cortesio et al., 2008). By using a model of melanoma cells, it has been revealed that Ca2 + oscillations act as a predisposing factor for invadopodia formation and activity through the involvement of STIM1 and ORAI1 channels, facilitating the assembly of invadosome precursors via activated Src, and regulating the focalized ECM degradation through the recycling of MT1-MMP (Sun et al., 2014). Moreover, within the Ca^2+^ microdomains the activation of Pyk2 initiates Src signaling cascade required for invasion (Lu et al., 2019). In glioblastoma multiforme cells, the expression and the activity of the major regulator of calcium-dependent signaling calmodulin correlate with the invasive capacity and invadopodia formation, by activating invadosome-associated proteins such as Src and NHE1 (Li et al., 2018). Moreover, this effect can be amplified by EGF, which promotes calmodulin translocation from the nucleus to the cytoplasm and its binding to Src and NHE1, further demonstrating the potency of the cooperation between different signaling converging on invadopodia. Beside these seminal works, the importance of calcium signaling is still far to be fully elucidated (Leverrier-Penna et al., 2020), and future investigations are warranted to determine the orchestrate molecular complex events linked to calcium signaling in regulating invadopodia and invasive behaviors.

## Conclusion

Tumor plasticity provides a new explanation for the mechanisms of invasion, metastasis, and recurrence, suggesting that interfering with the mediators of tumor plasticity is a becoming strategy to treat malignant tumors. During cancer progression, malignant cells must encompass different barriers requiring the dynamic interactions of cancer cells with the microenvironmental elements, including embedded stromal cells. Mechanical and biochemical interactions are associated with the generation of intracellular contraction forces that in turn restructure the surrounding TME. At the interface between cancer cells and metastatic processes, invadopodia are plastic structures with the ability to adapt their functions to respond to cellular and microenvironmental changes. Emerging evidence demonstrated that invadopodia are essential for cancer cell intravasation (Gligorijevic et al., 2014) and extravasation into specific microenvironments permissive for metastatic colony growth (Leong et al., 2014; Williams et al., 2019), as reported in preclinical mouse models, both affecting the efficiency of metastasis. Moreover, results from the analysis of tumor surgical specimens strongly support the existence of invadopodia inside human tumors, further underscoring the clinical relevance of invadopodia for human tumor biology (Chen Y.C. et al., 2019).

Significant advances have been made in understanding how invadopodia formation and activity triggered, for instance, by growth factor receptors are subject to different mechanisms of regulation, depending on the type of cells in which receptors are expressed and activated, as well as on the TME. We have learned that some molecular mechanisms and signaling pathways involved in invadopodia regulation are shared among different receptors while others are purely receptor-specific, cooperating with each other’s and with microenvironmental conditions. We now appreciate how the crosstalk between cancer cells and the biochemical and mechanically altered TME impacts invadopodia and tumor progression, since invadopodia sense and respond to the physical environmental properties through mechanotransduction processes, which in turn may impact the TME. These findings point to an important need to integrate the knowledge of how highly invasive cells could discern the multitude of biochemical and biomechanical cues, and extend our knowledge beyond those cues currently recognized to promote cancer progression.

More in depth studies are needed to appreciate overall of invadopodia regulation, to evaluate the relevance of individual mechanisms *in vivo* and to establish how signals from growth factor receptors cooperate with signaling from intracellular structures and the rest of the microenvironmental machinery, to provide an integrated perspective which can be translated in therapeutic approaches in cancer. As our understanding of biochemical and biomechanical cues encountered the cancer cells to control invadopodia formation/activity evolves, we will take us closer to find novel means to predict outcomes and evaluate therapeutic targets and approaches to control metastatic cancer, by blocking also invadopodia.

## Author Contributions

IM, VC, and AB contributed to manuscript writing. IM designed figures. LR wrote and edited the review. All authors contributed to the article and approved the submitted version.

## Conflict of Interest

The authors declare that the research was conducted in the absence of any commercial or financial relationships that could be construed as a potential conflict of interest.
